# Supplementary material for: The interactome of intact mitochondria by cross-linking mass spectrometry provides evidence for coexisting respiratory supercomplexes
Source: Mol Cell Proteomics. 2017 Dec 8;17(2):216–32. doi: 10.1074/mcp.RA117.000470 (PMC5795388; doi:10.1074/mcp.RA117.000470)
Supplement: Supplemental Data [file supp_RA117.000470_133922_0_supp_23978_fzffwf.zip › spectra_annotation/mito_spectra_annotation/86-1-3-1-13-1.pdf]

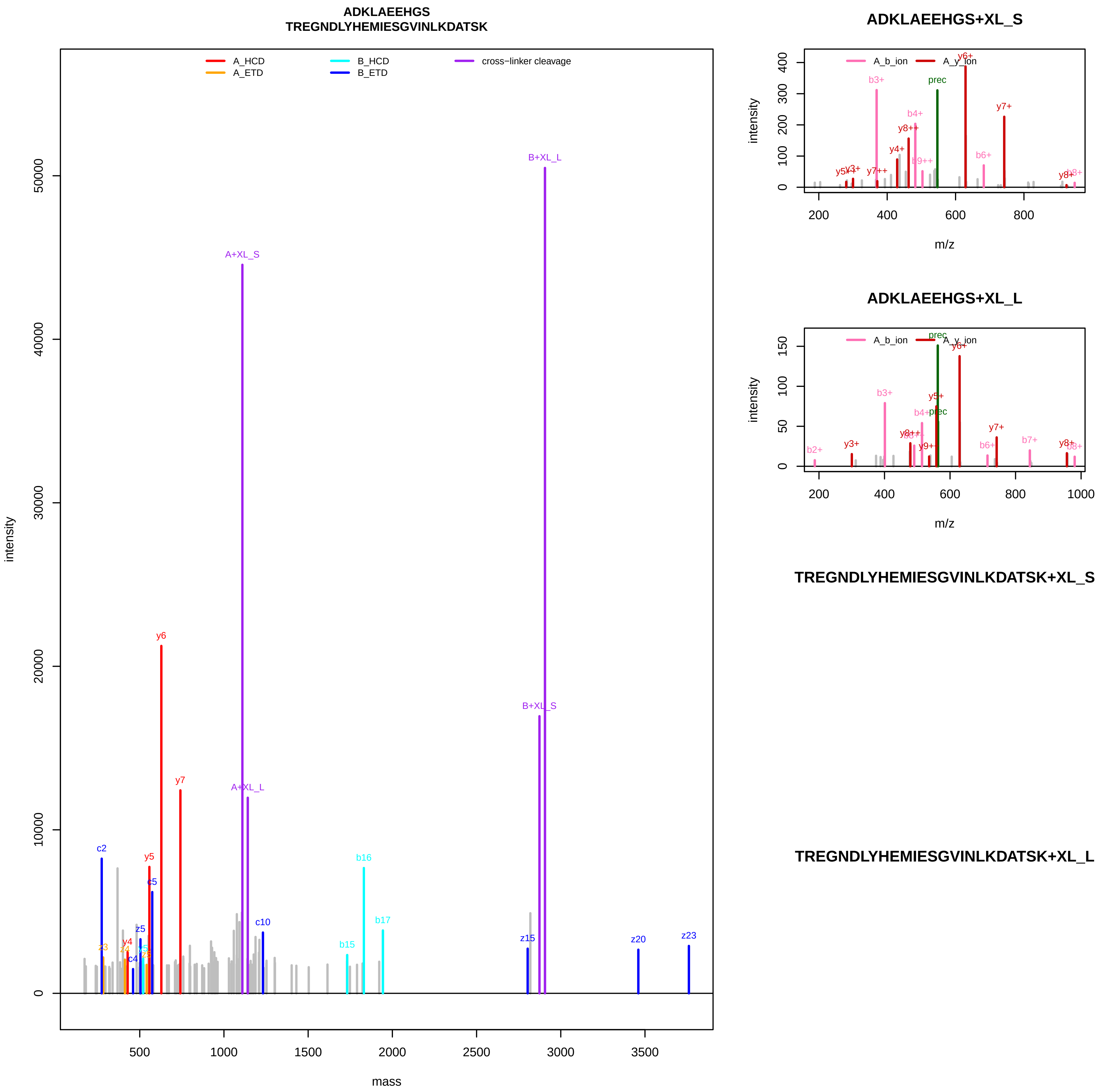

ADKLAEEHGS
TREGNDLYHEMIESGVINLKDATSK
A_HCD
A_ETD
B_HCD
B_ETD
cross−linker cleavage
intensity
mass
0
10000
20000
30000
40000
50000
500
1000
1500
2000
2500
3000
3500
B+XL_L
A+XL_S
y6
y7
A+XL_L
B+XL_S
c2
y5
c5
b16
b17
c10
z5
y4
z3
z4
c4
b15
z15
z20
z23
ADKLAEEHGS+XL_S
A_b_ion
A_y_ion
intensity
m/z
0
100
200
300
400
200
400
600
800
prec
b3+
y6+
y7+
b4+
y8++
y4+
b9++
b6+
y5++
y3+
y7++
y8+
b8+
ADKLAEEHGS+XL_L
A_b_ion
A_y_ion
intensity
m/z
0
50
100
150
200
400
600
800
1000
prec
y6+
b3+
y5+
b4+
y8++
y9+
y7+
b7+
b6+
y3+
b2+
y8+
b8+
TREGNDLYHEMIESGVINLKDATSK+XL_S
TREGNDLYHEMIESGVINLKDATSK+XL_L
